# Lack of specific T- and B-cell clonal expansions in multiple sclerosis patients with progressive multifocal leukoencephalopathy

**DOI:** 10.1038/s41598-019-53010-x

**Published:** 2019-11-12

**Authors:** Diego Bertoli, Alessandra Sottini, Ruggero Capra, Cristina Scarpazza, Roberto Bresciani, Luigi D. Notarangelo, Luisa Imberti

**Affiliations:** 1 Centro di Ricerca Emato-oncologica AIL (CREA), Diagnostic Department, ASST Spedali Civili, Brescia, Italy; 2 Department of Molecular and Translational Medicine, University of Brescia, Brescia, Italy; 3 Multiple Sclerosis Center, ASST Spedali Civili, Brescia, Italy; 4 Department of General Psychology, University of Padova, Padova, Italy; 5 Laboratory of Clinical Immunology and Microbiology, National Institute of Allergy and Infectious Diseases, National Institutes of Health, Bethesda, MD, United States

**Keywords:** Multiple sclerosis, Next-generation sequencing

## Abstract

Progressive multifocal leukoencephalopathy (PML) is a rare, potentially devastating myelin-degrading disease caused by the JC virus. PML occurs preferentially in patients with compromised immune system, but has been also observed in multiple sclerosis (MS) patients treated with disease-modifying drugs. We characterized T and B cells in 5 MS patients that developed PML, 4 during natalizumab therapy and one after alemtuzumab treatment, and in treated patients who did not develop the disease. Results revealed that: i) thymic and bone marrow output was impaired in 4 out 5 patients at the time of PML development; ii) T-cell repertoire was restricted; iii) clonally expanded T cells were present in all patients. However, common usage or pairings of T-cell receptor beta variable or joining genes, specific clonotypes or obvious “public” T-cell response were not detected at the moment of PML onset. Similarly, common restrictions were not found in the immunoglobulin heavy chain repertoire. The data indicate that no JCV-related specific T- and B-cell expansions were mounted at the time of PML. The current results enhance our understanding of JC virus infection and PML, and should be taken into account when choosing targeted therapies.

## Introduction

The JC virus (JCV) infects billions of people worldwide and maintains lifelong latency in the human body^1^. The infection usually is clinically silent. However, when the balance between the immune system and JCV is disrupted because of immunosuppression, the virus can reactivate, leading to progressive multifocal leukoencephalopathy (PML). Accordingly, PML occurs in individuals with severe and prolonged immunosuppression that affects T cells, as in patients with advanced HIV infection, and with hematological and solid malignancies. Furthermore, PML can also emerge as an adverse event of biologic therapies that alter the immune surveillance in the brain, such as natalizumab, a monoclonal antibody widely used to treat highly active multiple sclerosis (MS). Currently, PML secondary to treatment with natalizumab accounts for the majority of all cases of PML; in addition, PML has been observed also in few MS patients treated with other drugs, such as dimethyl fumarate and fingolimod^2^. To the best of our knowledge, no cases of PML attributable to ocrelizumab or teriflunomide have been described so far in MS patients, while a single case of a patient with clinical symptoms and features suggestive of PML has been reported during alemtuzumab therapy^3^. The extensive assessment on clinical, imaging and laboratory data strongly indicated that this was a typical case of PML as the American Academy of Neurology diagnostic criteria^4^ as well as the alternative diagnostic criteria^5^ were met. The evolution of JCV infection and the pathogenesis of PML is far from being fully understood. For example, it is not known how the immune response against JCV evolves over time. CD4+ and CD8+ cytotoxic T-cell recognition of viral antigens probably has a more important role than anti-JCV antibodies in limiting spreading of JCV infection^6^. JCV may infect the brain of immunocompetent individuals, but remains under a tight immunological control that involves brain-resident memory T cells, which rely on help from the peripheral recirculating CD4+ T cells^2^. These CD4+ T cells seem necessary to stimulate cytotoxic CD8+ T cells to clear JCV from the brain^7^. This can be the reason why prolonged treatment with immunosuppressive antibodies, such as natalizumab, increases the likelihood of PML insurgence^1^. In addition, B cells may also play a significant role in JCV control, principally due to their effects on T-cell responses through cytokine production.

Whether lymphocytes undergo specific modifications or show peculiar and common features across patients at the time of PML onset may help understand mechanisms of immune evasion and, in general, may enhance the understanding of anti-viral immune responses. In particular, the analysis of T-cell receptor (TR) and immunoglobulin heavy (IGH) repertoires of MS patients developing PML might allow the identification of T- and B-cell signatures specifically induced by JCV. In the past, several methods have been used for the analysis of TR beta (TRB) and IGH gene repertoires. These techniques include single strand conformational polymorphisms; singleplex or multiplex PCR of TRB and IGH gene rearrangements followed by third complementarity determining region (CDR3) length spectratyping/immunoscope and/or conventional sequencing; and flow cytometry-based staining with monoclonal antibodies specific for TRB variable (TRBV) chains. Using these approaches, several studies demonstrated skewed TR repertoires in peripheral blood or in cerebrospinal fluid (CSF) of MS patients^8–11^. In the periphery, clonal T-cell expansions appeared to be less prominent during natalizumab therapy^12,13^. On the contrary, TR repertoire restrictions were observed in the CSF of natalizumab-treated patients^13^. Interestingly, the same pattern was found in the peripheral blood of a patient who developed PML after 34 months of natalizumab therapy^14^. In patients who developed PML, new identical TR expansions in blood and CSF were observed following plasma exchange, and preceded the development of immune-reconstitution inflammatory syndrome^13^.

The previously used techniques only allow the examination of a small fraction of TRB and IGH rearrangements and thus do not enable comprehensive characterization of the T- and B-cell repertoires. These limitations were overcome by next generation sequencing (NGS) that, through the sequencing of millions of short templates, enables capturing also of infrequent clones^15^. This approach permits to establish accurately the diversity of the repertoire and to analyze the contribution of each individual clone in determining the overall repertoire complexity.

In MS patients, TRB and IGH sequencing by NGS has already been successfully employed to demonstrate the renewal of the repertoires after autologous stem cell transplantation as well as the dynamic recirculation of T and B cells between the periphery and the central nervous system^16–21^.

NGS could also allow to follow the modifications of TRB clonotypes (composed of highly frequent, also defined as dominant, as well as less frequent, defined as non-dominant, clones) and IGH changes upon PML insurgence. This might enhance our understanding on how immune repertoires evolve over time in treated patients, quantify the extent of repertoire modifications, and explore if patients share “public” immune response (i.e., clones with restricted TRB or IGH or CDR3 motifs shared identically by distinct individuals).

To investigate these issues, we performed a longitudinal analysis of T- and B-cell populations in samples of two MS patients obtained before natalizumab initiation, at 6 and 12 months post-therapy and, finally, at the time of PML onset. Samples obtained at the time of PML onset from three additional MS patients (two treated with natalizumab and one with alemtuzumab) were also analyzed, together with those of MS patients treated with natalizumab who did not develop PML.

## Results

### T- and B-cell subsets

**Figure 1:**
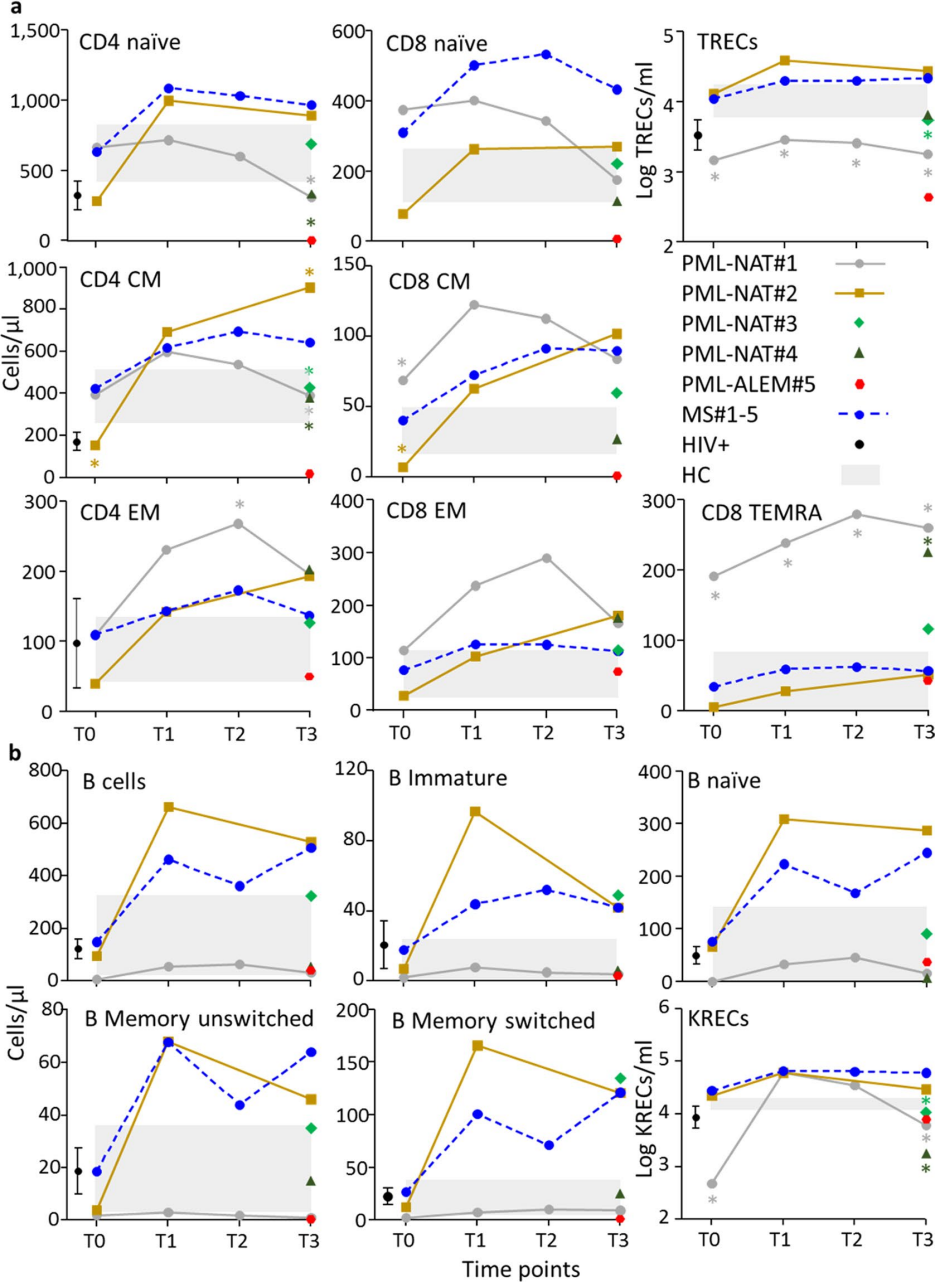
Characterization of T- and B-cell subsets. (**a**) Naïve lymphocytes were CD4+CD45RA+ and CD8+CD45RA+ cells, while central memory (CM) lymphocytes were cells with CD4+CD45RA-CCR7+ and CD8+CD45RA-CCR7+ phenotypes and effector memory (EM) lymphocytes were cells with CD4+CD45RA-CCR7- and CD8+CD45RA-CCR7- phenotypes. TEMRA were CD8+CD45RA+CCR7- cells. T-cell subtypes were given as absolute numbers (cells/µl) while TRECs (per ml) were reported after log transformation. (**b**) The B-cells were first gated for CD19 expression on lymphocytes, then analyzed for the expression of CD10 marker to identify CD19+CD10+ immature B cells and CD19+CD10- mature B cells. This last subset was examined for IgD and CD27 molecule expression in order to recognize IgD+CD27- naïve B cells, IgD+CD27+ unswitched memory B cells, and IgD-CD27+ switched memory B cells. B-cell subtypes were given as absolute numbers (cells/µl), while KRECs (per ml) were reported after log transformation. T- and B-cell subsets were analyzed at different time points in PML patients and MS controls (T0: samples prepared before natalizumab initiation; T1 and T2: at 6 and 12 months of therapy; and T3: at the time of PML. For details, see Table 2). For MS control patients, mean value for each immune parameter is reported. The color of the symbol is patient-specific. Light gray boxes represent the 99% confidence intervals of the means found in HC. Black symbol and bars indicate the values and the 99% confidence intervals for HIV+ patients. Values of PML patients outside these ranges are considered significant. With * are indicated the values statistically different from those of MS patients, in which are included MS#1 and MS#2, treated with natalizumab at the respective time point, as previously described^14^.

As expected, in the samples collected longitudinally from 5 natalizumab-treated controls patients who did not develop PML (MS#1-5), the number of T- and B-cell subsets with various phenotypes increased after the first 6 months of therapy (Fig. 1a). Most of T- and B-cell subsets were above the upper limit observed in HIV+ patients naïve for therapy and in age-matched healthy controls (HC). The lymphocytes of one natalizumab-treated patient who developed PML (PML-NAT#2) showed a similar pattern of T- and B-lymphocyte increase during the period of treatment, while in the other one (PML-NAT#1), the increase of most T- and B-cell subsets was followed by their decrease at the time of PML onset. In this last patient, at all-time points, the values of TR excision circles (TRECs), which are used as molecular marker for thymic output, were significantly under the lower limits established in HC and in MS#1-5, while CD8+ TEMRA cells were significantly higher than in these two control groups. At the time of PML onset, TRECs+ lymphocytes and CD8+ TEMRA cells were respectively at lower and higher level in 4 out 5 patients analyzed.

All B-cell subsets, including, total B cells, immature, naïve, memory switched and memory unswitched B lymphocytes and newly bone marrow-released B cells identified by the presence of K-deleting recombination excision circles (KRECs) were more represented in MS#1-5 and PML-NAT#2 than in HC (Fig. 1b). At PML onset, all PML patients, except PML-NAT#2, showed a number of KRECs+ lymphocytes lower than that of MS#1-5 and HC, while it was similar to that of HIV+ patients. The other B-cell subsets were heterogeneously represented.

### TR repertoire diversity measured by TRB CDR3 “conventional” spectratyping

**Figure 2:**
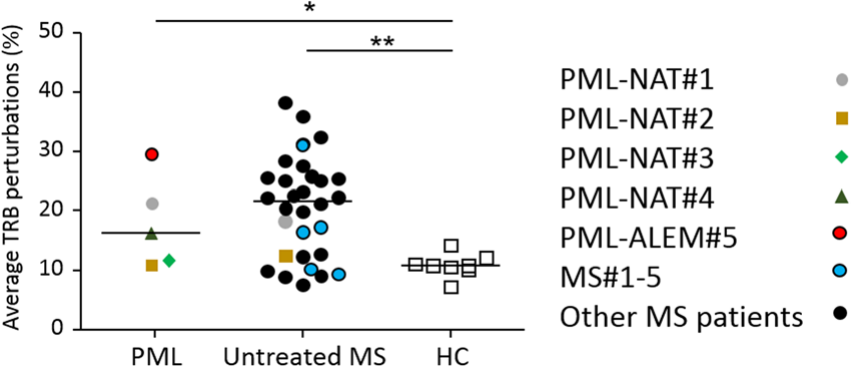
Percentages of TRB perturbations. Percentage of TRB distribution in PML patients (analyzed at the PML onset), MS patients who were untreated (including PML-NAT#1, PML-NAT#2 and MS#1-5 at T0) and HC. Each dot represents the global mean perturbation of the TRB repertoire in one patient. *P < 0.05; **P < 0.01.

The mean percentage of TRB perturbations, measured at the PML onset in the 5 PML patients, was similar to that of a group of 30 untreated MS patients (that included PML-NAT#1, PML-NAT#2 and MS#1-5 analyzed before natalizumab first infusion), but was significantly higher than in HC (Fig. 2). Deviations from the bell-shaped of TRB-CDR3 length distribution (Gaussian curve), which is characteristic of a TR repertoire diversification, were observed in samples prepared at the time of PML in PML-NAT#1, PML-NAT#3, PML-NAT#4, and PML-ALEM#5 (Supplementary Fig. S1). In particular, dominant peaks were present in the CDR3 length profiles of *TRBV10*, *TRBV20*, and *TRBV28* subgroups in PML-NAT#1; of *TRBV4* and *TRBV13* subgroups in PML-NAT#3; of *TRBV3*, *TRBV15*, and *TRBV16* in PML-NAT#4. In PML-ALEM#5, the CDR3 length profiles of most *TRBV* subgroups showed relevant peaks. However, skewing of the TRB repertoire was evident also before the onset of PML in the samples of PML-NAT#1, with the same dominant peaks observed in the sample at the PML onset. Furthermore, also in PML-NAT#2 at the time of PML onset, oligoclonal expansions were found in the *TRBV27* subgroup, but this *TRBV* subgroup was already skewed before and during natalizumab therapy.

### TRB and IGH diversity measured by NGS

#### Number of sequences and frequency of the more represented clones

Raw NGS data are available at the following link https://clients.adaptivebiotech.com/pub/bertoli-2019-sr or at the following 10.21417/DB2019SR.

A similar number of total productive templates and unique (that consider all identical sequences = 1) TRB and IGH rearrangements were obtained by NGS in samples prepared before natalizumab initiation (T0), during therapy (T1 and T2) and at the time of PML (T3) (Table 1). The only exception was PML-ALEM#5 in whom the total number of productive templates and of unique rearrangements was very low for both TRB and IGH. The degree of diversity was heterogeneous among groups of samples. The values of Shannon entropy were approaching 1 at all time points in MS#1 and MS#2, who never manifested PML, and in PML-NAT#2, indicating the greater diversity of both TRB and IGH repertoires. On the contrary, the lower Shannon entropy values found in samples of PML-NAT#1, PML-NAT#3, PML-NAT#4, and PML-ALEM#5 suggested the presence of less diversified TRB repertoires in 4 out 5 patients who developed PML.

**Table 1:** Summary of the number of TRB and IGH sequences obtained by NGS.

ID	Total templates	Total productive templates	Unique rearrangements	Normalized Shannon’s entropy^a^
**TRB**				
PML-NAT#1 T0	53,861	38,427	29,939	0.91
PML-NAT#2 T0	56,066	47,402	42,979	0.99
*mean*	***54***,***964***	***42***,***914***	***36***,***459***	
PML-NAT#1 T2	56,559	39,961	28,480	0.88
PML-NAT#2 T1	53,329	45,119	41,326	0.99
*mean*	***54***,***944***	***42***,***540***	***34***,***903***	
PML-NAT#1 T3	49,379	35,706	26,946	0.90
PML-NAT#2 T3	62,635	52,965	45,722	0.98
PML-NAT#3 T3	61,097	50,614	42,066	0.97
PML-NAT#4 T3	69,517	55,183	38,683	0.92
*mean*	***60***,***657***	***48***,***617***	***38***,***354***	
PML-ALEM#5 T3	4,215	3,558	1,611	0.80
MS#1 T0	58,146	46,832	44,188	0.99
MS#2 T0	50,309	41,285	37,300	0.99
*mean*	***54***,***228***	***44***,***059***	***40***,***744***	
MS#1 T2	46,644	37,723	34,847	0.99
MS#2 T2	51,264	42,501	38,482	0.99
*mean*	***48***,***954***	***40***,***112***	***36***,***665***	
**IGH**				
PML-NAT#1 T0	857	757	601	0.95
PML-NAT#2 T0	46,367	39,473	38,367	0.99
*mean*	***23***,***612***	***20***,***115***	***19***,***484***	
PML-NAT#1 T3	21,321	18,376	17,102	0.99
PML-NAT#2 T3	82,812	70,897	68,436	0.99
PML-NAT#3 T3	67,621	58,173	54,350	0.99
PML-NAT#4 T3	22,808	19,684	18,814	0.99
*mean*	***48***,***641***	***41***,***783***	***39***,***676***	
PML-ALEM#5 T3	5,566	4,784	4,527	0.99

^a^Normalized Shannon’s entropy (defined as 1-Pielou’s Evenness) is the index that measures the repertoire distribution. Values approaching 0 indicate a very skewed distribution of frequencies (i.e. more variation in abundance) and values approaching 1 indicate that every rearrangement is present at nearly identical frequency (i.e. less variation in abundance).

**Figure 3:**
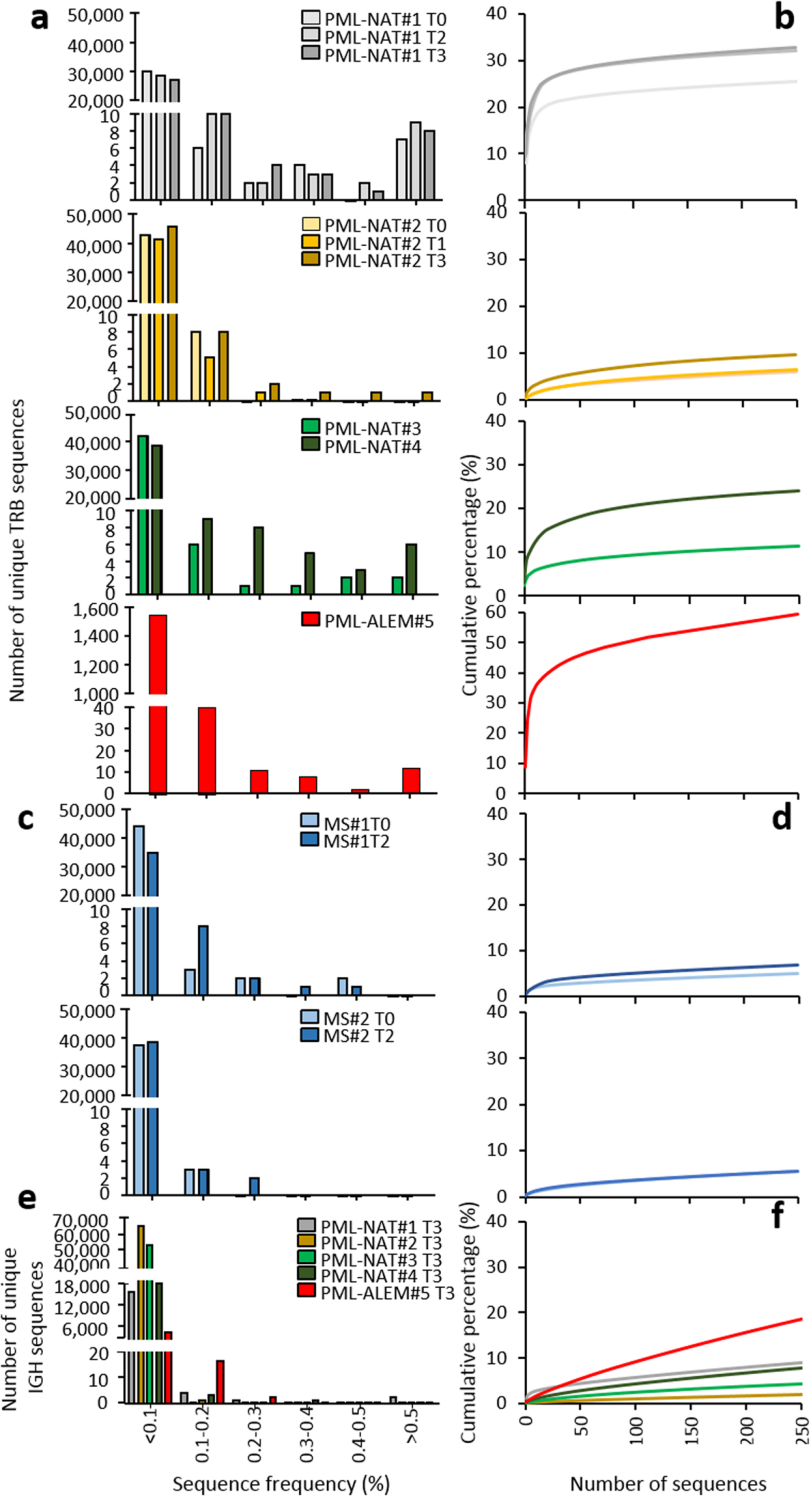
Frequency of TRB and IGH distribution and percentage of the 250 most frequent TRB and IGH sequences. (**a**) Frequency distributions and (**b**) cumulative percentage of the 250 most frequent TRB sequences detected by NGS before (T0), during (T1 and T2) natalizumab therapy and at PML development (T3) in PML-NAT#1 and PML-NAT#2; at the time of PML development in PML-NAT#3, PML-NAT#4 and PML-ALEM#5. (**c**) Frequency distributions and (**d**) cumulative percentage of the 250 most frequent TRB sequences in natalizumab-treated patients who did not develop PML (MS#1 and MS#2). (**e**) Frequency distributions and (**f**) cumulative percentage of the 250 most frequent IGH sequences in PML-NAT#1, PML-NAT#2, PML-NAT#3, PML-NAT#4 and PML-ALEM#5 at PML development (T3).

The vast majority of unique TRB and IGH sequences were present at a frequency below 0.1% in all analyzed samples, in both of patients that did and did not develop PML, while sequences with frequency >0.5% were found only in patients that developed PML (Fig. 3a). However, it should be noted that, in PML-NAT#1, a high number of sequences with a frequency >0.5% was already present in samples prepared before and during natalizumab therapy. The cumulative percentages of the 250 most frequent clones have been computed with the aim of exploring the extent of which the expanded clonotypes concur to the total TRB repertoires. These sequences constituted 60%, 30%, and 20%, of the total TRB repertoires present at PML onset in PML-ALEM#5, PML-NAT#1, and PML-NAT#3, respectively (Fig. 3b). The expanded clonotypes accounted for only the 10% of the total TRB repertoire in PML-NAT#2 (Fig. 3b), and for even a lower fraction in MS#1 and MS#2 (Fig. 3c, d). Few expanded (frequency > 0.5%) IGH clonotypes were found at PML onset (Fig. 3e), but the 250 most frequent clones reached about 20% of the total IGH repertoire in PML-ALEM#5 while were less than 10% in the other patients (Fig. 3f).

#### TRB and IGH CDR3 length distribution

The CDR3 amino acid length distribution of TRBV -Diversity (D)-Joining (J) and IGHV-Joining (J) gene rearrangements was calculated according to IMGT (The international ImMunoGeneTics information system^®^; http://www.imgt.org). The CDR3 was considered to start at amino acid 105, after the conserved cysteine of the *V* gene, and to extend until position 117, before the conserved phenylalanine and tryptophan in the *J* gene. When the analysis of CDR3 length distribution was performed first singularly for each patient, we observed that none of CDR3 profiles of total and unique sequences fit to a Gaussian distribution curve (data not shown; D’Agostino & Pearson normality test: P < 0.05). The more skewed CDR3 length profiles were found in samples obtained at PML onset (Supplementary Fig. S2a). Then, this analysis was performed in three groups of patients: the first included all sequences obtained from PML-NAT#1 and PML-NAT#2 before and during natalizumab therapy (T0 and T1/T2 = group I); the second comprised sequences from PML-NAT#1, PML-NAT#2, PML-NAT#3, PML-NAT#4 and PML-ALEM#5 at the PML onset (group II); and the third those from the MS#1 and MS#2 (T0 and T2 = group III). The mean TRB CDR3 amino acid lengths did not differ significantly in these three groups either for total (12.2 ± 1.2 in group I, 12.1 ± 1.6 in group II, and 12.3 ± 1.6 in group III; P = NS) or for unique sequences (12.5 ± 1.6 in group I, 12.4 ± 1.6 in group II, and 12.4 ± 1.6 in group III; P = NS). However, consistent with the perturbations of TRB repertoires obtained by evaluation of spectratyping data, the CDR3 profiles of total sequences were not found to fit to a Gaussian curve, suggesting the presence of perturbed TRB repertoires (Supplementary Fig. S2b; D’Agostino & Pearson normality test: P < 0.05). By contrast, the IGH CDR3 length distribution in the 5 PML patients fitted the Gaussian distribution curve (D’Agostino & Pearson normality test: P = NS). CDR3 composed by 14 amino acids were the most represented (Supplementary Fig. S2c).

### *TRBV*, *TRBJ*, *IGHV* and *IGHJ* gene usage

**Figure 4:**
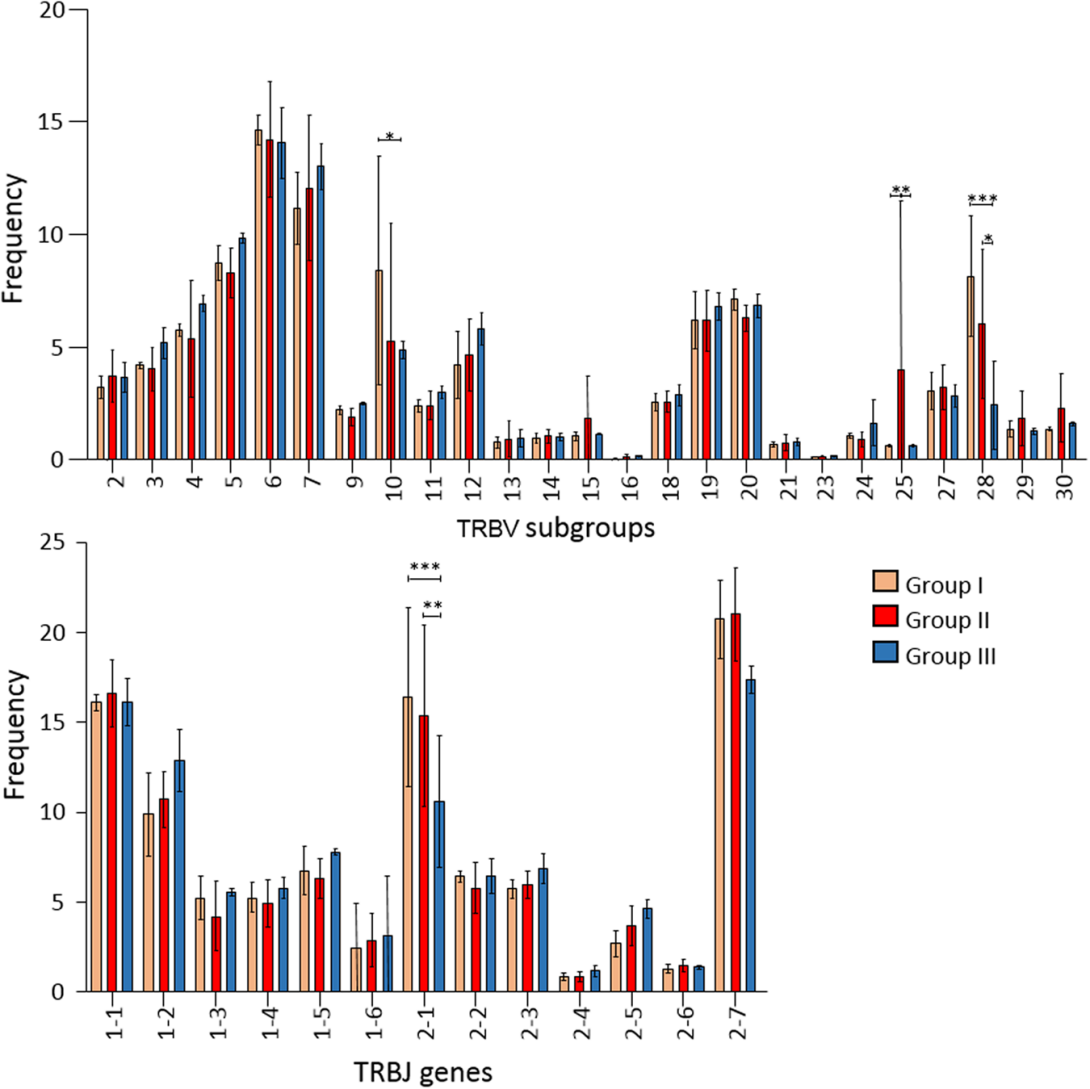
Usage of *TRBV* subgroups and *TRBJ* genes in patient groups. Relative frequency of *TRBV* subgroups and *TRBJ* genes usage in total sequences of group I (PML-NAT#1 and PML-NAT#2 at T0 and T1/T2), group II (PML-NAT#1, PML-NAT#2, PML-NAT#3, PML-NAT#4 and PML-ALEM#5 at T3), and group III (MS#1 and MS#2 at T0 and T3). *P < 0.05; **P < 0.01; and ***P < 0.001.

Rearrangements involving *TRBV1*, *TRBV8*, *TRBV17*, *TRBV22*, and *TRBV26* subgroups were excluded from the analysis of gene usage since, according to IMGT, they were not translated into functional protein. Those that utilized *TRBV*6, *TRBV10* and *TRBV28* subgroups were predominantly represented in all samples of PML-NAT#1, those utilizing preferentially *TRBV6* (18% of the sequences) and *TRBV25* subgroups in PML-ALEM#5 (Supplementary Fig. S3a). In these two patients, the *TRBJ* genes preferentially involved in the rearrangements were *TRBJ2*-*1* (21% of PML-NAT#1 sequences) and TRBJ2-*7* (Supplementary Fig. S3b). The usage of *TRBV* subgroups and *TRBJ* genes did not differ in longitudinal samples prepared at different time points in PML-NAT#1 and PML-NAT#2 and MS#1 and MS#2. Finally, there was no evidence of common preferential *TRBV*-*TRBJ* rearrangements: *TRBV10*-*TRBJ2*-*1* pairing was observed in 11.4% of the sequences of PML-NAT#1 at T2 and T3, while *TRBV6*-*TRBJ2*-*7* rearrangements accounted for 10% of pairing in PML-ALEM#5. Due to the presence of dominant clones in individual patient, the usage of *TRBV10*, *TRBV25*, *TRBV28*, and *TRBJ2*-*1* subgroups was significantly different also when analyzed in patient group I, II and III (Fig. 4). Similarly, *TRBV6* and *TRBV7* subgroups and *TRBJ1*-*1*, *TRBJ2*-*1*, and *TRBJ2*-*7* genes were the most abundantly rearranged ones in the three groups, but a significant different usage was observed only for *TRBJ2-1* gene (Fig. 4).

The *IGHV3* and *IGHV4* subgroups, and *IGHJ6* and *IGHJ4* genes were preferentially used in samples obtained at the time of PML. No differences were observed in samples of PML-NAT#1 and PML-NAT#2 obtained before the first natalizumab infusion (Supplementary Fig. S4). *IGHV3* preferentially rearranged with *IGHJ4* (*IGHV3*-*IGHJ4* pairing ranging from 26.2% in PML-NAT#4 to 16.7% in PML-NAT#3) and *IGHJ6* (*IGHV3*-*IGHJ6* ranging from 23.4% in PML-NAT#3 to 15.1% in PML-ALEM#5). However, while the percentage of *IGHV3*-*IGHJ6* sequences of PML-NAT#1 remained unmodified in the samples obtained before the beginning of natalizumab therapy and at the onset of PML, the *IGHV4*-*IGHJ6* pairing was 22.1% before the therapy and 7.8% at the time of PML onset. Finally, *IGHV3*-*IGHJ3* pairing increased from 3.4% to 6.0% from T0 to T3.

### Analysis of amino acid composition of *TRB* and *IGH* sequences

TRB sequences were considered identical if they shared the same V and J regions and included the same CDR3. Using this strategy, the 5 PML patients were found to share only 2 identical TRB sequences at the time of PML onset, while 44 identical sequences were identified when only the four natalizumab-treated PML ones were considered. Of these, two sequences were also observed in samples obtained before PML onset, and none was present in the database containing published sequences of untreated MS patients^22^.

“Public PML” clonotypes were defined as identical CDR3 sequences shared by at least two patients’ samples obtained at the time of PML onset, which were not present before PML onset or in MS#1 and MS#2 at any time points, or in databases of public CDR3 clonotypes of HC^23^ and of patients with other diseases^22,24^. In the 5 samples obtained at PML onset, 770 public clonotypes were found; within these clonotypes the most frequent amino acid was glycine, that was dominantly present from positions 109 to 112 of CDR3 (Supplementary Fig. S5a). However, very similar sequence pattern was observed in the 586 public clonotypes identified in MS#1 and MS#2 using the same criteria adopted for identifying PML public clonotypes (Supplementary Fig. S5b).

**Figure 5:**
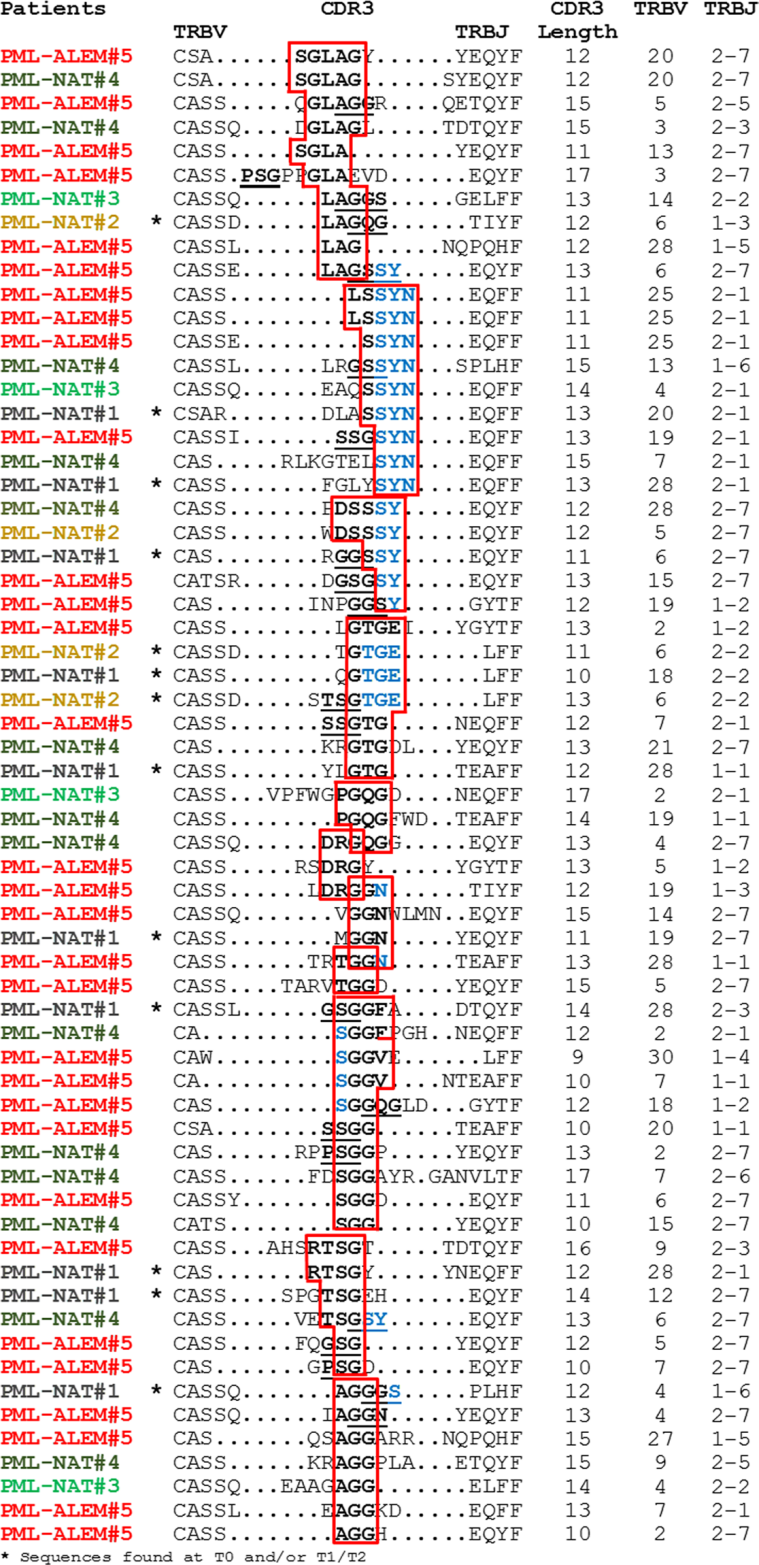
TRB CDR3 amino acidic composition of expanded clones. CDR3, TRBV and TRBJ region definitions of the sequences of expanded clones was done according to IMGT nomenclature. Common amino acid stretches were evidenced in boxes or were underlined.

Expanded amino acids motifs were found in 183 clones that may not share V and J regions, may have different CDR3 length and not complete identity in the amino acid composition of CDR3, but were present at a frequency >0.1% in the 5 patients at the PML onset. They were highly represented in PML-ALEM#5 (n. 73), PML-NAT#4 (n. 45), and PML-NAT#1 (n. 34). The other two patients have a number of expanded motifs (n. 18 in PML-NAT#2 and n. 13 in PML-NAT#3) similar to those of patients under natalizumab therapy (n. 18 in MS#1 and n. 8 in MS#2). However, of the 52 expanded motifs of PML-NAT#1 and PML-NAT#2 identified at PML onset, only two were not present in the samples obtained at other time points. Sixty-three of 183 expanded motifs included common CDR3 stretches that were not found in the expanded sequences with a frequency >0.1% of MS#1 and MS#2 (Fig. 5). Among them, three stretches of 5 amino acids were detected, two (SGLAG and LSSYN) in two different clones of PML-ALEM#5 and one (DSSSY) in two clones identified one in PML-NAT#4 and one in PML-NAT#2. Shorter versions of the above-mentioned stretches, formed by 4 (GLAG and SSYN) or three (GLA, LAG and SSY) amino acids were included in several other expanded clones. Other common stretches formed by 4 (GTGE, SGGF, SGGV and RTSG) amino acids or by their short version formed by three amino acids (GTG, SGG and TSG) were identified in several other clones. Some of the amino acids at the right positions of these stretches were codified by the initial part of *TRBJ* genes (in light blue in the Fig. 5).

The 5 patients did not share identical IGH sequences at any time points. Since public clonotypes are usually extremely rare in IGH chains^25^, and because no common identical IGH sequences were identified in our patients, “public PML” IGH clonotypes have not been searched.

## Discussion

Some viruses can persist in balance with and for the life of their immunocompetent host, often without causing any symptoms^26^. However, if the delicate virus-host equilibrium is altered, a critical threshold can be reached that may lead to an unrestrained viral replication and subsequent infectious episodes, as observed in aging or when cellular immunity is weakened by therapies^27^. For instance, cellular immunity is strongly influenced by biological disease-modifying anti-rheumatic drugs that increase the susceptibility to varicella zoster virus reactivation in rheumatoid arthritis patients^28^. In addition, despite the different mechanism of action, MS therapies associated with PML can eventually lead to an immunosuppression within the CNS that increases the probability of reactivation of the latent virus, thereby facilitating productive infection of glia by JCV^29^.

As PML is an uncommon event, insight on T- and B-cell phenotypic changes at the time of disease onset is still very limited. Previous data suggest that the containment of JCV infection is critically dependent on specific CD4+ and CD8+ lymphocyte response^30^. For examples, PML that occurs in HIV-infected patients, knowing to have an impaired thymopoiesis, and therefore less likely to develop a robust JCV-specific cellular immune response^31^, benefits from highly active antiretroviral therapies that induce a recovery of anti-JCV T-cell immunity^32–34^. Antiretroviral therapy, that stimulates thymic output, participates to immune responses against viral pathogens by providing a TR repertoire with broad antigenic specificity^35,36^. An immune system well-replenished with new T cells, with many and different specificities, can be able to recognize all viral antigens and develop an anti-viral specific immune response. Previous observations reported that also MS patients might exhibit reduced thymic output and reduced TR repertoire diversity^37,38^. These features have been previously reported also in a case of natalizumab-related PML^14^ and in one alemtuzumab-treated patient who developed PML^3^. Here, we provide evidence that thymic output was also impaired in two additional patients at PML onset. The reduced production of new cells was counterbalanced by the peripheral expansion of memory lymphocytes and, in particular, of CD8+ TEMRA cells. This was particularly evident in the patient who received alemtuzumab, in whom, despite the profound lymphopenia induced by the treatment, the memory T-cell number was within the normal range. Consistent with the increase of memory cells, oligoclonal T-cell expansions, demonstrated by the presence of several dominant peaks in the CDR3 profiles, were detected in all PML patients. However, the expanded clonotypes found at the PML onset were already present before treatment initiation and persisted during therapy. In addition, the global TRB perturbations were similarly present in patients who did or did not develop PML.

To obtain more precise and complete information on T cells at PML onset, a NGS analysis of the TRB CDR3 repertoire, at the level of individual clone, was performed. This may lead to a better understanding of T-cell responses against viruses as, despite the potentially vast TR repertoire diversity, similar restrictions are commonly identified in response to persistent infections. Two examples are the Epstein-Barr virus-^39^ and HIV-^40^ related infections. These TR repertoire restrictions include expansion of clones with identical V(D)J rearrangements, the preferential usage of *TRBV* or *TRBJ* genes without conserved amino acids in the CDR3 region, and the selection of identical amino acids (up to 5) or ‘motifs’ in the CDR3. These characteristics may even be shared among individuals, thus defining the so-called “public” T-cell responses, which allow different individuals to develop similar antigen-specific TR specificities against some ubiquitous pathogens.

We found that PML patients showed a slightly perturbed TRB repertoire compared to MS patients; moreover, expanded clonotypes, i.e. with a productive frequency >0.5%, were present only in PML patients, especially at disease onset. However, the analysis of single TRB CDR3 sequences revealed the absence of specific clonotypic expansions at PML development, because the most expanded clones found at the time of PML were already present before natalizumab initiation and during therapy. Therefore, in our patients, we did not identify potentially JCV-specific clones as those described after *invitro* stimulation with JCV peptides of brain-infiltrating CD4+ T cells of one natalizumab-related PML patient^41^. Since these data were obtained starting from single time point samples of individual patients and using peptides belonging to the same portion of JCV, the obtained T-cell clones might represent only a selected and small part of the possible TR specificities used to mount an efficient immune response to JCV. In addition, since public clones should be present only in patients with a common HLA background, the identification of a few public PML clonotypes is not unexpected. HLA alleles may influence the susceptibility and response to JCV infection. For instance, brain-derived T-cell clones recognize different peptides of JCV major capsid protein in the context of multiple HLA-DR and HLA-DQ molecules. The ability to use multiple restriction elements to recognize the same and/or different peptides, increases binding avidity and ensures *in vivo* activation even when one restriction element is poorly expressed. The sustained antigenic stimulation can lead to the selection of those T clones that have the best binding capacity for the immunodominant JCV epitope^42^. In addition, HLA alleles associated with risk for developing autoimmune diseases appear to be inversely related with PML^29^. For instance, HLA-DRB1*15, the most well-established susceptibility allele for MS risk, and HLA-DRB1*13, which is negatively related with various autoimmune diseases, may confer protection against JCV^29^.

Even if we found that all 5 PML patients shared sequences with identical CDR3 amino acidic composition, these sequences were not exclusive for the PML onset, as they were also present in the two MS patients who did not develop the disease and/or in HC. Furthermore, public PML clonotypes, defined as sequences exclusively found at PML onset and shared by at least two patients, displayed a very similar amino acidic composition when compared to public MS clonotypes identified in the two patients who did not develop PML.

However, common and exclusive amino acid motifs were observed within expanded clones obtained at PML onset, with the 5 amino acid long motifs (serine-glycine-leucine-alanine-glycine and leucine-serine-serine-tyrosine-asparagine), together with their shorter three or 4 amino acid long versions mostly represented within expanded clonotypes. Nevertheless, it is worth noting that several clones with leucine-serine-serine-tyrosine-asparagine motif included amino acids codified by a portion of *TRBJ2*-*1* that, accordingly, was the most used *TRBJ* gene in all sequences. Therefore, it is likely that these motifs were not specifically induced by JCV. Other motifs frequently observed were glycine-threonine-glycine and serine-glycine-glycine that were previously described in TRB sequences obtained from JCV peptide-induced T-cell clones^41^, but they cannot be consider as JCV specific due to the low number of amino acids of the motif. In addition, no specific amino acid hydrophobicity was identified at position 6 and 7 of the CDR3 that is known to strongly promote the development of self-reactive TRs in mice^43^.

The current data do not indicate a common usage of *TRBV* or *TRBJ* genes or preferential *TRBV*-*TRBJ* pairings when PML developed and a preferential use of *TRBV6*, *TRBV7*, *TRBJ2*-*7* subgroups and *TRBJ2*-*1* genes was observed in all patients. While the *TRBJ2*-*7* gene was preferentially used in MS patients and in adult and infant HC analyzed in previous studies^44,45^, the *TRBV6* subgroup was not the most frequently utilized *TRBV* gene in these controls.

B lymphocytes also play a role in PML pathogenesis because they may serve both as a JCV reservoir and as vectors for viral dissemination within the CNS^30^. Furthermore, although JCV replication in the CNS appears not to be linked to anti-JCV antibody responses, B cells may influence T-cell response by producing cytokines^30^. A detailed analysis showed that natalizumab treatment leads to a significant increase in the numbers of memory and marginal zone-like B cells, while the number of naïve B cells is not influenced^46^. Consistent with this, we observed a reduced production of new KREC+ B cells in three natalizumab-related PML and in the single patient with alemtuzumab-induced PML. The other B-cell subsets were heterogeneously represented during PML, but this was an expected finding, as the proportion of these cells in peripheral blood is incredibly variable also among HC^47^.

Analysis of the IGH rearrangements suggested a diverse repertoire in all samples obtained at the PML onset, with the only exception of PML-ALEM#5, in whom IGH clonotypes with a frequency >0.5% constitute about the 20% of the total IGH repertoire. Furthermore, no common IGH sequences were identified at the PML onset. *IGHV3* was by far the most frequently utilized *IGHV* subgroup in the 5 PML patients, as previously found also in HC^45,48^. However, differently from HC, in whom predominant usage of the *IGHJ4* gene is observed^46^, in our PML patients, two *IGHJ* were almost equally represented, with *IGHJ6* being the most abundant, followed by *IGHJ4*. Finally, despite the preferential usage, rearrangement and joining of certain *V* and *J* genes, a no dominant or common features of IGH sequences were observed at PML onset.

A limitation of this study is that the analysis was limited to peripheral blood, while analysis of lymphocytes present in CSF or in the brain may provide more valuable information for a better understanding of natalizumab-associated PML^7,42^. Indeed, it has been reported that in these patients the CSF T-cell pool is not only diminished quantitatively, but it is also characterized by a reduced TR heterogeneity^13,49–51^. In addition, due the inherent descriptive nature of this study, the limited cohort (only MS-treatment associated PML), and the lack of the T- and B-repertoire characterization in other hosts, it is unclear whether our findings represent a feature of JCV infection or merely the response of immunocompromised hosts. However, restricted TR repertoires in the periphery and CSF may critically weaken CNS immune surveillance and helps to explain loss of control of chronic infection due to JCV in a proportion of patients.

Altogether, our data demonstrated that, although there are several alterations of T-and B-cell subsets, as well as different TR and IGH repertoire, no specific or common features were identified in peripheral blood at the PML onset. In particular, data obtained with longitudinal samples indicated that there were not specific changes of the dominant T and B clonotypes from natalizumab therapy initiation and PML onset and that, apparently, no JCV-related specific T- and B-cell expansions were mounted at the time of PML. In this way our results is in line with the hypothesis that JCV infection would thus represent a model of tissue-dependent immune tolerance, being JCV tolerated in a peripheral organ, as is the kidney, but under tight control in the brain. This enhance our understanding of the latent JCV infection and PML and should be taken into consideration when dealing with development of targeted therapies.

## Material and Methods

### Patients and samples

We investigated the peripheral T- and B-cell composition of 5 MS patients who developed natalizumab-related (PML-NAT#1, PML-NAT #2, PML-NAT #3 and PML-NAT #4) and alemtuzumab-related (PML-ALEM#5) PML. They developed PML respectively after 34, 25, 16, 56, and 15 months of therapy (T3). Their clinic and demographic features are reported in Table 2. PML was suspected based on clinical presentation and magnetic resonance images findings. A lumbar puncture always followed the clinical and radiological suspect of PML and the diagnosis was verified by JCV DNA detection in the CSF and was consistent with a diagnostic accuracy of “definitive” according with the PML consensus statement of the American Association of Neurology^4^.

**Table 2:** Patient characteristics and available samples.

ID	sex	Age (years)	Previous therapy	Last therapy	Age (years)		Disease duration (years)		JCV-DNA copies/ml	EDSS	Available samples - time points			
											T0^a^	T1	T2	T3^b^
		at MS diagnosis			at the last therapy start	at PML onset	at the last therapy start	at PML onset		at PML onset	(months of therapy)			
PML-NAT#1^c^	F	21	IFNβ 1a (30 µg/w; 22 and 44 µg 3 times/w) MTX (120 mg/m^2^)	NTZ	39	41	19	20	24	7.5	0	6	12	34
PML-NAT#2	F	24	IFNβ 1a (30 µg/w; 22 and 44 µg 3 times/w)	NTZ	27	28	3	4	73	3.5	0	6	—	25
PML-NAT#3	F	35	IFNβ 1a (22 and 44 µg 3 times/w)	NTZ	47	48	12	14	13	3.5	—	—	—	16
PML-NAT#4	F	9	IFNβ 1b	NTZ	29	34	20	25	10	5	—	—	—	56
PML-ALEM#5	F	23	IFNβ 1a (22 and 44 µg 3 times/w); fingolimod, GA, DMF	Alem	30	31	8	9	197^d^	na	—	—	—	15
MS#1	M	23	IFNβ1a (22 µg 3 times/w)	NTZ	37	—	17	—	—	—	0	6	12	15
MS#2	F	15	IFNβ 1a (30 µg/w; 22 and 44 µg 3 times/w)	NTZ	17	—	7	—	—	—	0	6	12	27
MS#3	M	31	IFNβ 1a (30 µg/w and 22 µg 3 times/w); MTX (110 mg/m^2^)	NTZ	37	—	12	—	—	—	0	6	12	23
MS#4	F	29	IFNβ 1a (44 µg/3 times/w); IFNβ 1b; MTX (90 mg/m^2^); GA (20 mg/day)	NTZ	41	—	15	—	—	—	0	6	12	32
MS#5	F	31	IFNβ 1a (44 µg 3 times/w)	NTZ	32	—	5	—	—	—	0	6	12	24

^a^Pre-natalizumab or alemtuzumab therapy; ^b^PML diagnosis (the number correspond to the months of natalizumab/alemtuzumab therapy); ^c^previously descrided^14^; ^d^cut-off: 150 copies/ml. Alem: alemtuzumab; DMF: dymethyl fumarate; GA: glatiramer acetate; ID: Identification name; IFNβ: interferon beta; MTX: mitoxantrone; na: not available; —: not applicable; NTZ: natalizumab; w- week.

Peripheral blood mononuclear cells were isolated by Ficoll-Hypaque density gradient centrifugation at the time of PML onset. Samples from PML-NAT#1 and PML-NAT#2 were also obtained longitudinally, before natalizumab start (T0) and during the therapy (T1, T2). Longitudinal samples from 5 MS patients under natalizumab therapy (MS#1-5), who did not develop PML, obtained from the group of patients previously collected and described^14^, were used as controls, together with samples obtained from 16 HIV+ patients naïve for therapy, used as positive control for viral infection, and with age-matched HC (which number could be different in the different experimental procedures). The timing of sample collection is indicated in Table 2. The study was approved by the Ethics Committee of Brescia (approval no. NP2713 of 24/05/2017 and 11/10/2018) and was conducted according to the principles expressed in the Declaration of Helsinki and its later amendments. Written informed consent was obtained from all patients.

### Real-time PCR for TRECs and KRECs, and cytofluorimetric characterization of T- and B-cell subpopulations

TRECs and KRECs were measured, as previously described^52^, by duplex real-time PCR (7500 Fast real-time PCR system; Applied Biosystems Foster City, CA) in DNA extracted from peripheral blood mononuclear cells with QIAamp DNA Blood Mini Kit (Qiagen, Valencia, CA). Both were expressed per ml of blood because their number was corrected for lymphocyte plus monocyte count in 1 ml of blood.

T- and B-cell subsets were determined by cytofluorimetric analysis (BD FACSCanto II, BD Biosciences, San Jose, CA). Briefly, phycoerythrin anti-CD3, allophycocyanin-H7 anti-CD4, phycoerythrin-Cy7 anti-CD8, fluorescein isothiocyanate anti-CD45RA (BD Pharmingen, Heidelberg, Germany), peridinin-chlorophyll protein-Cy5.5 anti-CCR7 (BioLegend, San Diego, CA) monoclonal antibodies were used for T-cell subsets characterization. For B-cell subpopulation identification, peridinin-chlorophyll protein-Cy5.5 anti-CD19, phycoerythrin-Cy7 anti-CD10, fluorescein isothiocyanate anti-IgD, and phycoerythrin anti-CD27 monoclonal antibodies (BD Biosciences) were used. Data were analyzed using FACS Diva software (BD Biosciences).

### TRB CDR3 length spectratyping

Total RNA was prepared using NucleoSpin^®^ RNA kit (Macherey-Nagel GmbH & Co. KG Duren Germany). The first strand of complementary DNA, synthesized using random examers and TaqMan Reverse transcription reagents (Applied Biosystems), was immediately utilized for 5 *TRBV* gene multiplex PCR that allow detection of the 23 functional *TRBV* subgroups^53^. Fluorescent PCR products were electrophoresed on a 3500 genetic analyzer (Applied Biosystems). Distribution of fragment lengths, number of detectable peaks per *TRBV* subgroups, and area under the curve were calculated by Gene Mapper software (Applied Biosystems). The global perturbation of the TRB repertoire of a single patient was expressed as mean percentage of TRB perturbations, obtained by the single *TRBV* perturbations calculated with the generalized Hamming distance method. This implies the “subtraction” from the CDR3 length distribution of each *TRBV* of a patient, the mean Gaussian-like CDR3 length distribution, obtained by analyzing a “reference group” composed of 8 HC. When a *TRBV* subgroup was not represented (no detectable peaks), the condition of maximal perturbation was reached, and its value was arbitrarily set to 100%.

### TRB and IGH sequencing by NGS

*TRB* and *IGH* sequencing was performed at Adaptive Biotechnologies (Seattle, WA, USA) using the immunoSEQ platform^54^ and the survey level assay. After CDR3 region amplification from genomic DNA by two-step multiplex PCR approach and library sequencing, reads were processed to reduce amplification and sequencing bias and were quantified against a set of synthetic TR CDR3 sequence analogs.

These synthetic inline controls (that include every known V-J pairings) were used in each PCR reaction. Since they were present at known concentrations in the initial mix, the amplification and sequencing efficiency was monitored, allowing to know how many reads correspond to a single template in each reaction. Moreover, using this synthetic immune receptor repertoire, PCR biases were minimized and computationally removed after sequencing^55^.

Raw data processing and analysis were performed with the ImmunoSEQ Analyzer software. Out of frame sequences and sequences with stop codons within the CDR3 were removed. *TRB* and *IGH* gene designations are in accord with IMGT nomenclature.

The Skylign software^56^, using observed count method option, was used to identify amino acidic composition and its frequency at each position of the CDR3.

### Statistical analysis

Values of immune parameters were considered statistically different from those of natalizumab-treated patients and HC when they were outside the confidence intervals of their respective means. In order to account for multiple comparisons, Bonferroni corrected p value = 0.01 was employed and therefore the 99% confidence intervals were calculated for each immune parameters tested (TREC and KREC confidence intervals were calculated after log transformation of data).

Mean TR perturbation percentages were analyzed by nonparametric methods (Mann-Whitney and Wilcoxon test, with Bonferroni corrected p values) and CDR3 length distribution by D’Agostino & Pearson normality test. The differences between the means of CDR3 length distributions were calculated by Kruskal-Wallis test and Dunn’s post-hoc test. *TRBV*, *TRBJ*, *IGHV* and *IGHJ* gene usage were analyzed by ANOVA with Bonferroni correction.

## Supplementary information

Supplementary Data
