# Supplementary material for: Lack of specific T- and B-cell clonal expansions in multiple sclerosis patients with progressive multifocal leukoencephalopathy
Source: Sci Rep. 2019 Nov 12;9:16605. doi: 10.1038/s41598-019-53010-x (PMC6851145; doi:10.1038/s41598-019-53010-x)
Supplement: Supplementary file 1 — Supplementary Data [file 41598_2019_53010_MOESM1_ESM.pdf]

Lack of specific T- and B-cell clonal expansions in multiple sclerosis patients with progressive multifocal leukoencephalopathy

Diego Bertoli[1,2], Alessandra Sottini[1], Ruggero Capra[3], Cristina Scarpazza[3,4], Roberto Bresciani[2], Luigi D. Notarangelo[5], Luisa Imberti[1,*]

[1] Centro di Ricerca Emato-oncologica AIL (CREA), Diagnostic Department, ASST Spedali Civili, Brescia, Italy

[2] Department of Molecular and Translational Medicine, University of Brescia, Brescia, Italy

[3] Multiple Sclerosis Center, ASST Spedali Civili, Brescia, Italy

[4] Department of General Psychology, University of Padova, Padova, Italy

[5] Laboratory of Clinical Immunology and Microbiology, National Institute of Allergy and Infectious Diseases, National Institutes of Health, Bethesda

* Correspondence should be addressed to L.I. (email: luisa.imberti@asst-spedalicivili.it)

Supplementary Figures

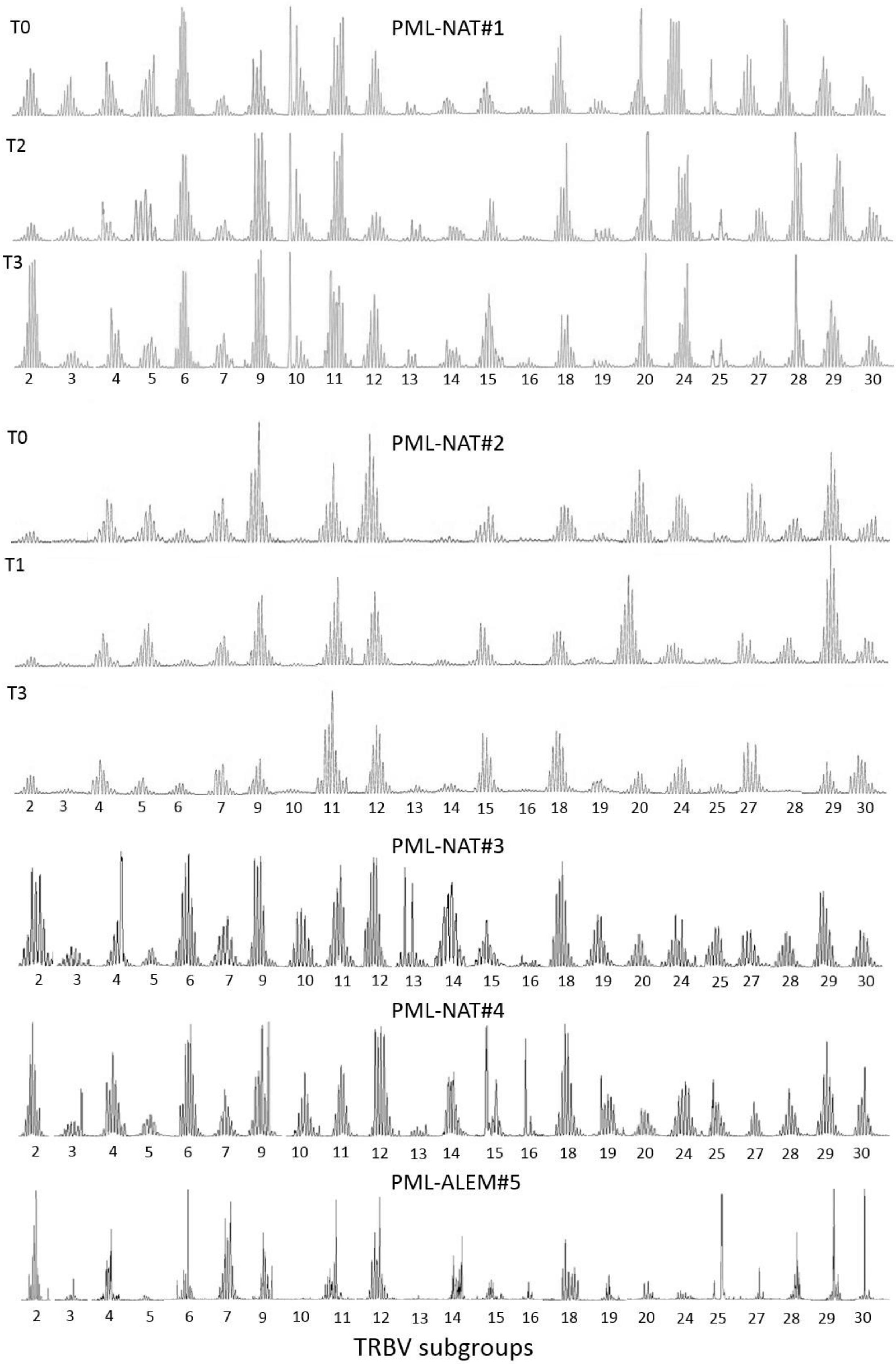

PML-NAT#1
T0
T2
T3
2 3 4 5 6 7 9 10 11 12 13 14 15 16 18 19 20 24 25 27 28 29 30
PML-NAT#2
T0
T1
T3
2 3 4 5 6 7 9 10 11 12 13 14 15 16 18 19 20 24 25 27 28 29 30
PML-NAT#3
2 3 4 5 6 7 9 10 11 12 13 14 15 16 18 19 20 24 25 27 28 29 30
PML-NAT#4
2 3 4 5 6 7 9 10 11 12 13 14 15 16 18 19 20 24 25 27 28 29 30
PML-ALEM#5
2 3 4 5 6 7 9 10 11 12 13 14 15 16 18 19 20 24 25 27 28 29 30
TRBV subgroups

Supplementary Figure S1. TRB CDR3 spectratyping of PML patients

CDR3 length distribution for each one *TRBV* subgroups is performed at different time points (for details see Table 2) through RNA extraction, multiplex PCR and capillary electrophoresis.

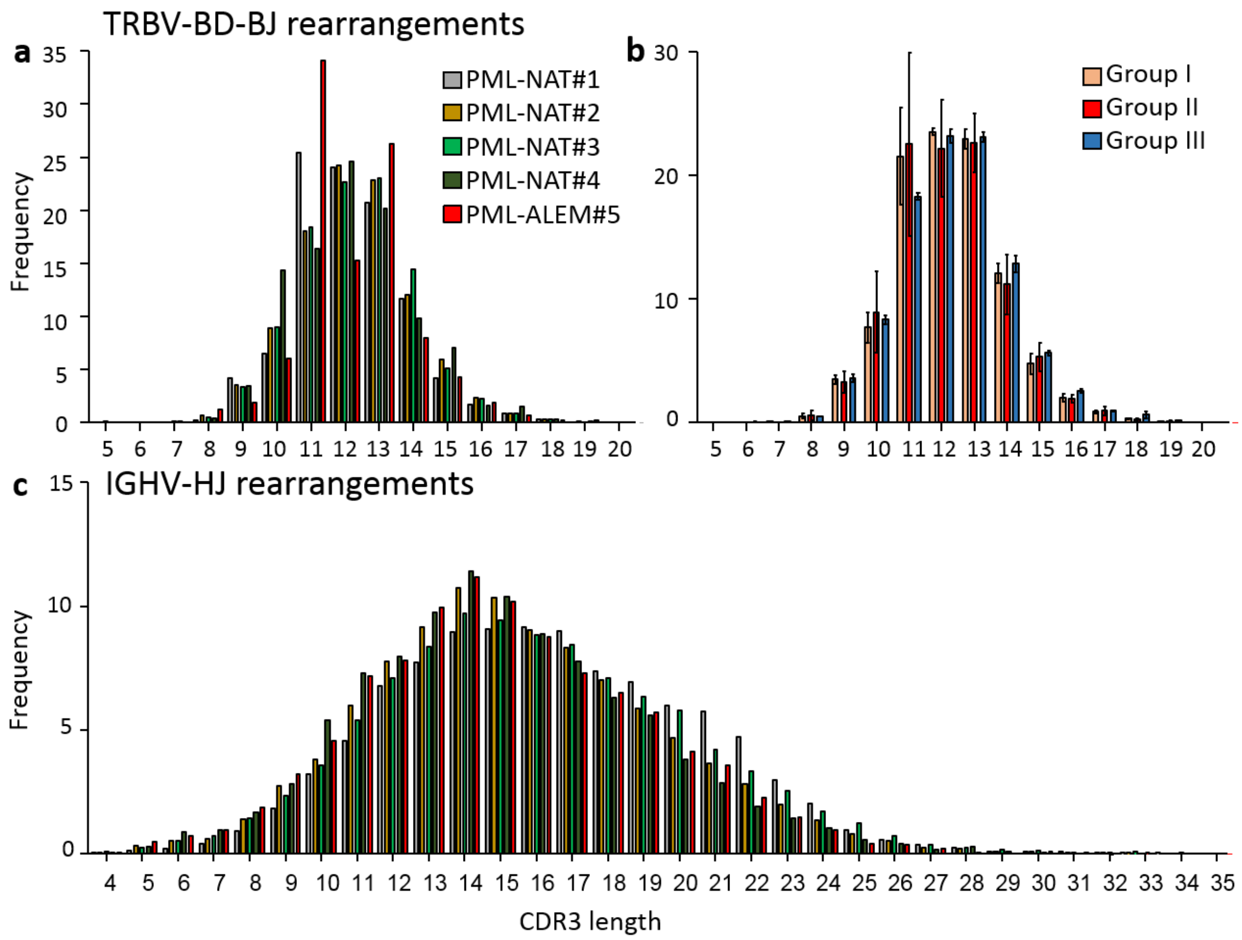

Supplementary Figure S2. Characteristics of the CDR3 of TRBV-BD-BJ and IGHV-HJ rearrangements

Distribution of the CDR3 length of TRBV-BD-BJ rearrangements a) in PML-NAT#1, PML-NAT#2, PML-NAT#3, PML-NAT#4 and PML-ALEM#5 and b) in group I (PML-NAT#1 and PML-NAT#2 at T0 and T1/T2); group II (PML-NAT#1, PML-NAT#2, PML-NAT#3, PML-NAT#4 and PML-ALEM#5 at T3); and group III (MS#1 and MS#2 at T0 and T2). c) Distribution of the CDR3 length of IGHV-HJ in PML-NAT#1, PML-NAT#2, PML-NAT#3, PML-NAT#4 and PML-ALEM#5.

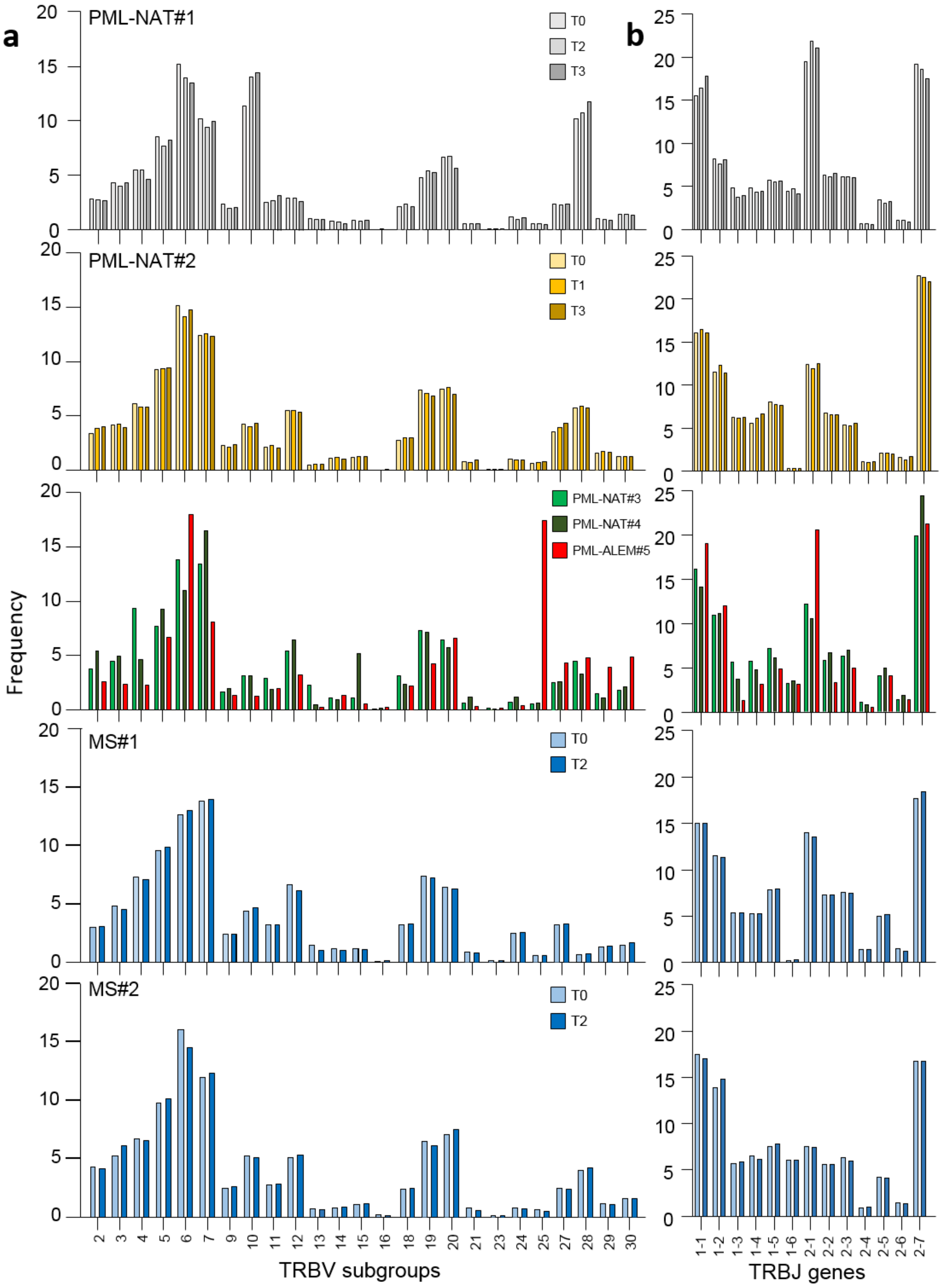

a
b
PML-NAT#1
T0
T2
T3
PML-NAT#2
T0
T1
T3
PML-NAT#3
PML-NAT#4
PML-ALEM#5
MS#1
T0
T2
MS#2
T0
T2
Frequency
TRBV subgroups
TRBJ genes

Supplementary Figure S3. Usage of *TRBV* subgroups and *TRBJ* genes at different time points

Relative frequency of usage a) of *TRBV* subgroups and b) of *TRBJ* genes in total sequences of PML-NAT#1 and PML-NAT#2 at T0, T1/T2 and T3, PML-NAT#3, PML-NAT#4 and PML-ALEM#5 at T3, and MS#1 and MS#2 at T0 and T2.

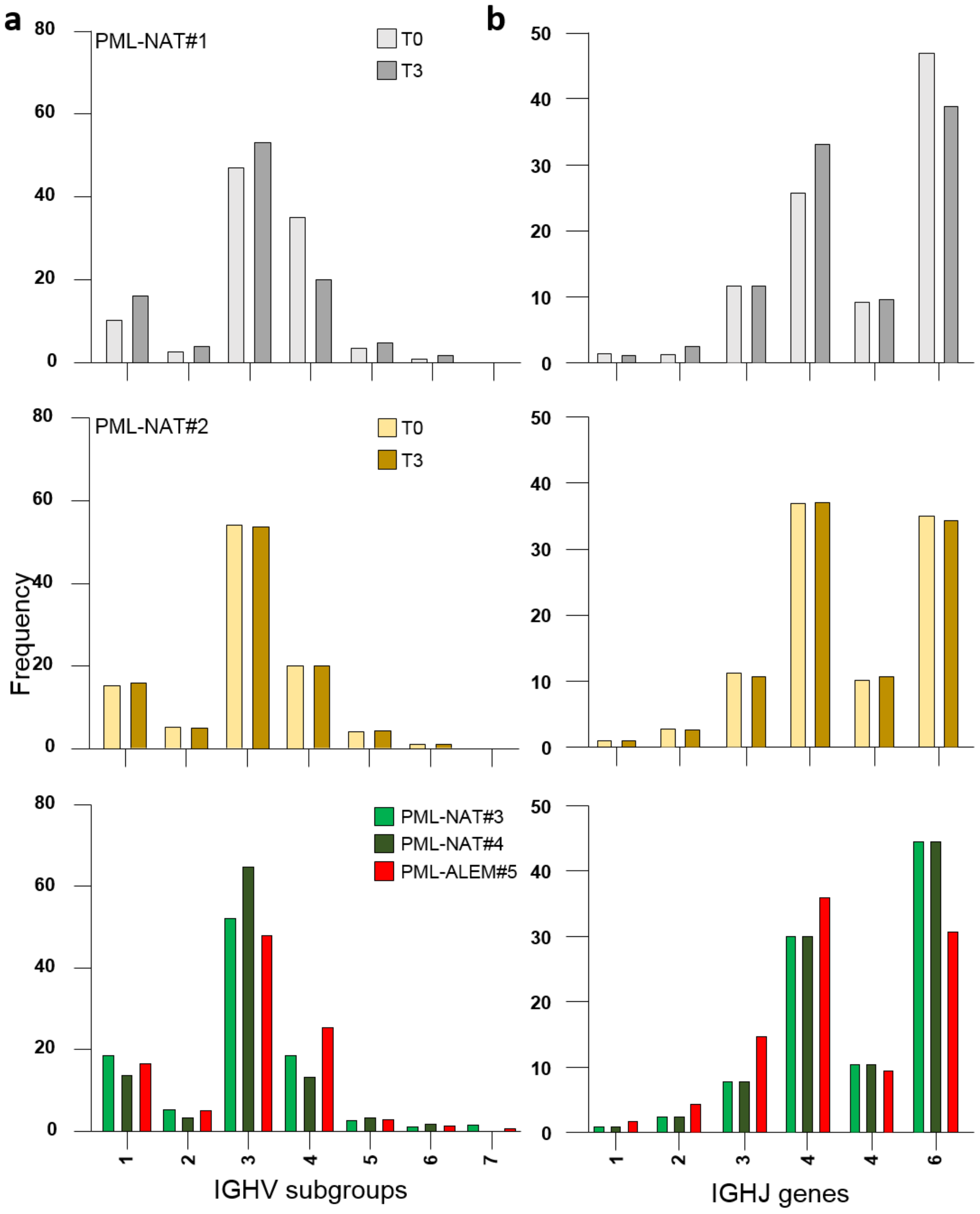

Supplementary Figure S4. Usage of *IGHV* subgroups and *IGHJ* genes at different time points

Relative frequency of usage a) of *IGHV* subgroups and b) of *IGHJ* genes in total sequences of PML-NAT#1 and PML-NAT#2 at T0 and T3, and PML-NAT#3, PML-NAT#4 and PML-ALEM#5 at T3.

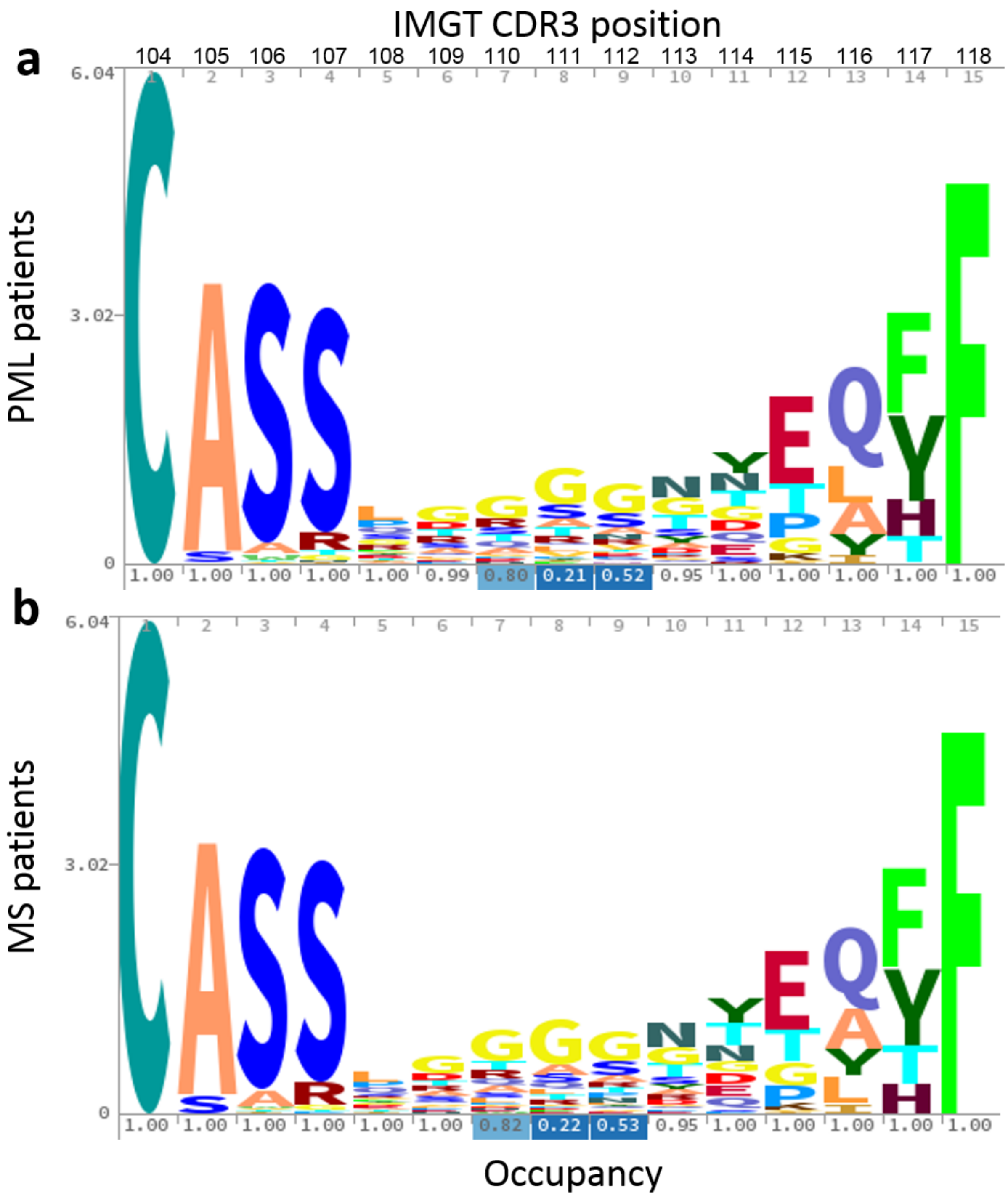

Supplementary Figure S5. CDR3 amino acid usage in public TRB clonotypes.

CDR3 amino acid composition in clonotypes of a) PML and of b) MS patients (was calculated using observed count method option of the Skylign software[56]. The relative size of each amino acid symbol is proportional to its frequency. Occupancy reported on the x-axis indicates the probability of observing a letter in that position.
